# Neoisoastilbin Ameliorates Acute Gouty Arthritis via Suppression of the NF-*κ*B/NLRP3 Pathway

**DOI:** 10.1155/2023/7629066

**Published:** 2023-02-14

**Authors:** Yan Wang, Xiaoxi Zhang, Changyu Xu, Yanjing Yao, Chenxi Wu, Wenjing Xu, Fenfen Li, Daozong Xia

**Affiliations:** ^1^School of Pharmaceutical Sciences, Zhejiang Chinese Medical University, Hangzhou 310053, China; ^2^Academy of Chinese Medical Sciences, Zhejiang Chinese Medical University, Hangzhou 310053, China

## Abstract

Acute gouty arthritis (AGA) is an acute inflammatory disease, whose occurrence and development mechanism are associated with inflammatory reaction of joint tissue. This study investigated the role of neoisoastilbin (NIA) in the treatment of AGA and explored the underlying mechanisms. C57BL/6 mice underwent intraarticular injection of monosodium urate (MSU) to establish an AGA model in vivo. Enzyme-linked immunosorbent assay, histopathological hematoxylin-eosin staining, western blotting, and other methods were used to observe the therapeutic effects of NIA on AGA and investigate the role of the NF-*κ*B/NLRP3 pathway in the treatment. We found that NIA effectively reduced MSU-induced joint swelling and inflammatory cell infiltration in a concentration-dependent manner. NIA also significantly reduced interleukin-1*β* (IL-1*β*), interleukin-6 (IL-6), and tumor necrosis factor-*α* (TNF-*α*) levels as compared with the respective values in the model mice group. In addition, administration of NIA significantly mitigated the phosphorylation of NF-*κ*B-related proteins (IKK*α*, NF-*κ*B, and I*κ*B*α*) and the expression of NLRP3-related proteins (NLRP3, caspase-1, and ASC) in MSU-induced joint tissues. In conclusion, our research indicated that NIA significantly improved AGA, and its underlying mechanism was achieved by simultaneously inhibiting the NF-*κ*B/NLRP3 pathway and the expression of inflammatory factors. This research preliminarily suggested the potential role of NIA in the treatment of AGA.

## 1. Introduction

The incidence of gout is on the rise worldwide, with gouty arthritis accounting for the most prevalent type [1, 2]. Its acute onset is an acute inflammatory response caused by the deposition of monosodium urate (MSU) microcrystals in the periarticular tissues, which seriously affects the quality of life of patients [3]. Acute gouty arthritis (AGA) is responsible for an increased risk of developing hypertension, chronic kidney disease, obesity, diabetic kidney stones, myocardial infarction, heart failure, and stroke [4].

A critical role of MSU-induced inflammation in the pathogenesis of AGA has been reported [5]. MSU remains in an ionic state under physiological conditions and precipitates in the peripheral tissues under excessive concentrations of blood uric acid. After MSU precipitation, monocytes drift towards the periarticular tissues and become activated. Once activated, NLRP3 transduces the recognition signal to the adapter apoptosis-relatedspeck-like protein (ASC) to facilitate activation of caspase-1, thereby stimulating the release of multiple inflammatory factors including interleukin-1*β* (IL-1*β*), interleukin-6 (IL-6), and tumor necrosis factor-*α* (TNF-*α*) [6, 7]. Furthermore, inflammatory factors cause severe pain and joint destruction of patients [8, 9]. Therefore, inhibiting MSU-induced inflammation could be an effective therapeutic strategy for AGA.

At present, nonsteroidal anti-inflammatory drugs, colchicine, and corticosteroids are by far the most efficacious anti-AGA agents that exert their therapeutic effects by inhibiting inflammation [10, 11]. However, these drugs cause side effects and toxicity, which restrict their application [12–14]. These limitations have led to the search for new therapeutic drugs.

Neoisoastilbin (NIA), a kind of flavonoid, holds antioxidant and anti-inflammatory effects, and its molecular structure resembles that of astilbin [15, 16]. It has been reported that *Rhizoma smilacis glabrae* water extract, which contains neoisoastilbin, effectively alleviates potassium oxonate- and MSU-induced hyperuricemia and gout in mice [17]. In addition, the total flavonoids of *Smilax glabra* Roxb., which are mainly astilbin, neoastilbin, isoastilbin, and neoisoastilbin, have therapeutic activity against hyperuricemia [18]. Nevertheless, no studies in the literature have explored the efficacy of NIA in AGA. In the present study, we established an AGA model using intra-articular MSU injection in mice and evaluated the protective effects and potential mechanisms of NIA on the experimental mice.

## 2. Method

### 2.1. Materials

Neoisoastilbin with a purity of over 85% was prepared in the laboratory according to the procedures described previously [15]. Briefly, the *Smilax glabra* Roxb. was air-dried, ground, and extracted three times with ethanol/water (60 : 40, v/v) at 80°C for 2 h each; then, the extract was filtered through Whatman No. 1 filter paper, and the ethanol from the extract was removed under vacuum. Then, the residue was dissolved in distilled water and further fractionated with *n*-hexane, ethyl acetate, and *n*-butanol. Then, isolation and preparation of neoisoastilbin were executed on Prep 150 LC system (Waters, Milford, MA, USA) consisted of a Waters 2545Q preparative pump equipped with a Waters 2489 UV/Visible Detector, Waters Fraction Collector III, Waters 2707 Automatic Sampler, and a preparative column (Waters Sunfire Prep C18 OBDTM 250 × 19 mm, 5 *μ*m). Monosodium urate (MSU, Lot: BC8R7559) was purchased from Sigma-Aldrich (USA). Colchicine tablets (Lot: 17EN) were purchased from Kpc Pharmaceuticals Inc. (Kunming, China) for animal experiments. RIPA lysis buffer (Lot: 01408/20307), phosphatase inhibitor cocktail (Lot: 01385/40247), protease inhibitor cocktail 2-amino-2-(hydroxymethyl)-1, 3-propanediol (Lot: 01392/60333), BCA protein concentration detection kit (Lot: 50206), and other commonly used reagent consumables were purchased from Cowin (Jiangsu, China). IL-1*β* ELISA Kit (Lot: MM-0040M1), TNF-*α* ELISA Kit (Lot: MM-0132M1), and IL-6 ELISA Kit (Lot: MM-0163M1) were purchased from Enzyme Immunity (Jiangsu, China). Primary antibodies against NLRP3 (Lot: 3), caspase-1 (Lot: 1), ASC (Lot: 2), p-IKK*α* (Lot: 19), p-NF-*κ*B (Lot: 16), p-I*κ*B*α* 4 (Lot: 18), IKK*α* (Lot: 5), NF-*κ*B (Lot: 9), I*κ*B*α* (Lot: 10), *β*-actin (Lot: 15), and Histone H3 (Lot: 9) were obtained from Cell Signaling Technology (USA). NE-PER™ Kit (Lot: QE217174) was purchased from Thermo Scientific (USA). Western ECL substrate (Lot: 1178) was purchased from Bio-Rad (USA).

### 2.2. Mouse Model of AGA and NIA Treatment

Six-week-old male C57BL/6 mice (22.5 ± 2 g) (Shanghai, China, certificate number: SCXK 2017-0005) were used for inducing the mice model of AGA. Animal experiments were approved by the Animal Care and Use Committee of Zhejiang Chinese Medical University (permission number: SYXK 2018-0012). Mice were cultured in the SPF laboratory animal room at constant temperature and humidity with a 12 h dark/light cycle with adequate access to diet and water.

After adapting to the environment for one week, the C57BL/6 mice were randomly divided into five groups: (a) control group (Con); (b) MSU treatment group (MSU); (c) MSU with colchicine treatment group (Col, 1 mg/kg); (d) MSU with low-dose NIA treatment group (NIA-L, 25 mg/kg); (e) MSU with high-dose NIA treatment group (NIA-H, 50 mg/kg). All mice were given the appropriate drugs for 7 days. On the 6th of intragastric administration, the joints of the mice in the normal group were injected with 0.025 mL of physiological salt solution, and the joints of the remaining mice were injected with 0.025 mL of MSU at a concentration of 50 mg/mL to induce the AGA model. At the end of the experiment, all mice were executed, and joints were removed for subsequent experiments.

### 2.3. Measurement of Mouse Ankle Joint Swelling

Before measurement, a line was drawn at the upside of the ankle joint as fiducial marks demarcating the ankle joint areas to be analyzed. The ankle joint was inserted into a specific measuring cylinder filled with water. The syringe was pushed to allow the water surface to reach the marked level of the labeled ankle and data were recorded. The toe volumes of mice were measured by a toe volume measuring device before modeling and 2, 4, 6, 10, and 24 h after modeling.

### 2.4. Hematoxylin-Eosin Staining

The joint tissues were fixed in 4% paraformaldehyde at room temperature. Next, the tissues were paraffin embedded and cut into 5 *μ*m slices. After removal of paraffin and endogenous peroxidase, the tissues were stained with hematoxylin and eosin. Finally, the sections were sealed with ultra-thin cover glass, and the histopathological changes of the different joint tissues were observed under an optical microscope.

### 2.5. Enzyme-Linked Immunosorbent Assay

The joint tissues were ground in liquid nitrogen and immersed in PBS. Tissue homogenates were collected and centrifuged at 4°C for 10 min under the condition of 12000 rpm. The supernatant was taken to determine the levels of IL-1*β*, IL-6, and TNF-*α* according to the kit instructions.

### 2.6. Western-Blotting Analysis

The joint tissues were ground in liquid nitrogen and lysed using RIPA lysis buffer with a 1% phosphatase inhibitor cocktail and a 1% protease inhibitor cocktail for 30 min. Then, tissue homogenates were centrifuged at 12000 rpm for 10 min, and the supernatant was taken to determine the protein concentration. Meanwhile, the nuclear proteins and cytoplasmic proteins were isolated according to the procedure of the NE-PER™ Kit. After that, the same amounts of proteins were separated by 10% sodium dodecyl sulfate-polyacrylamide gel electrophoresis (SDS-PAGE), and subsequently separated proteins were transferred to PVDF membranes. The membranes were then blocked in 5% nonfat milk for 2 h and incubated with the different primary antibodies overnight. Subsequently, the membranes were incubated with the HRP-labeled goat antimouse or rabbit IgG antibodies for 2 h. The detection was performed by the chemiluminescence method. The results of western blotting were obtained using the software Image *J*.

### 2.7. Statistical Analysis

In this study, the results were analyzed by one-way analysis of variance (ANOVA), followed by multiple comparisons with the Dunnett test using the statistical software of SPSS 24.0 or a two-tailed unpaired Student's *t*-test using GraphPad Prism 8.0. All calculated data were expressed as mean ± standard deviation. In this study, *p* < 0.05 was considered statistically significant, and *p* < 0.01 was considered very statistically significant.

## 3. Results

### 3.1. NIA Alleviates MSU-Induced AGA in Mice

**Figure 1:**
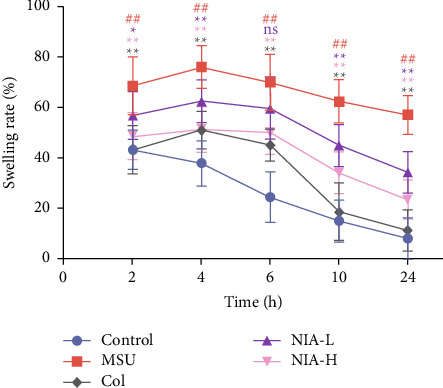
NIA markedly ameliorated joint swelling in the mouse model of MSU-induced AGA. The changes in joint swelling indices of mice 2, 4, 6, 10, and 24 h after modeling. The results shown are the means ± SD of ten independent experiments; ^#^*p* < 0.05 or ^##^*p* < 0.01 compared with the Con group; ^*∗*^*p* < 0.05 or ^*∗∗*^*p* < 0.01 compared with the MSU group.

The joint swelling is a principal marker of AGA.  Figure 1 shows the swelling changes in all groups. The rate of joint swelling in Con mice was consistently reduced over the period that was monitored. Mice in the MSU group exhibited a higher rate of joint swelling, which was significant over the measured time range (2–24 h) compared to the Con group. The swellings reached their peak after 4 h and then showed a slight decrease within 4–24 h. Consistent with the previous study [19], the Col group significantly reduced MSU-induced joint swelling, as did the NIA low-dose and high-dose groups. The result indicated that both NIA doses showed preventive effects on joint swelling, and such effects were dose-dependent.

**Figure 2:**
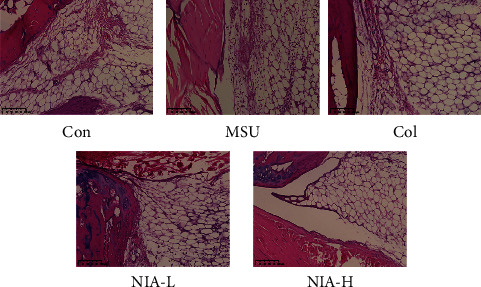
NIA improved inflammatory cell infiltration in the joint. Hematoxylin-eosin staining of the joints. Each picture shows a representative example of joint tissues from a different group. Magnification: ×20.

We also evaluated the histological status of all groups by hematoxylin-eosin staining. As shown in Figure 2, the MSU group had extensive inflammatory cell infiltration. However, infiltration of inflammatory cells was considerably improved in the NIA-treated group.

### 3.2. NIA Inhibited Inflammatory Factor Expression in Mice

**Figure 3:**
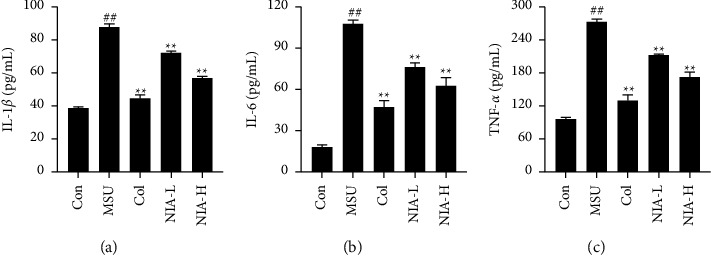
NIA inhibited levels of IL-1*β*, IL-6, and TNF-*α* in joints. (a) Levels of IL-1*β* in mice joints. (b) Levels of IL-6 in mice joints. (c) Levels of TNF-*α* in mice joints. The results shown are the means ± SD of ten independent experiments; ^#^*p* < 0.05 or ^##^*p* < 0.01 compared with the Con group; ^*∗*^*p* < 0.05 or ^*∗∗*^*p* < 0.01 compared with the MSU group.

In order to assess the role of NIA in AGA, the levels of inflammatory factors (including IL-1*β*, IL-6, and TNF-*α*) in joint tissues were measured. As shown in Figure 3, the levels of IL-1*β*, IL-6, and TNF-*α* in the joints of mice were significantly increased in the MSU group, which were 2.3, 6.0, and 2.8 times higher than those in the Con group, respectively. This indicated that the accumulation of MSU crystals in the joints significantly promoted the expression of inflammatory factors, and interestingly, NIA significantly modulated the levels of inflammatory factors, and the effect of the high-dose group was better than that of the low-dose group.

### 3.3. NIA Modulated NF-*κ*B/NLRP3 Pathway in Mice

**Figure 4:**
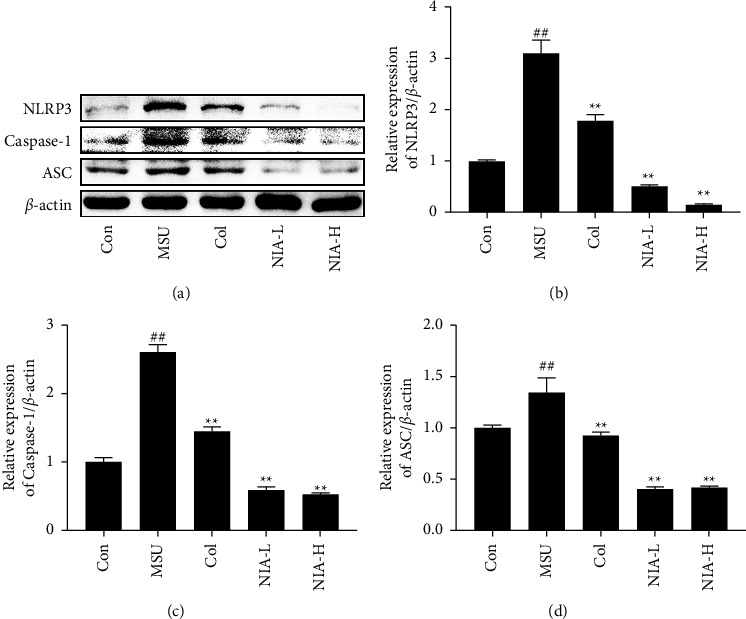
NIA inhibited NLRP3 inflammasome in mice joints. (a) Western blotting analysis of the NLRP3 pathway. The relative protein expression levels of (b) NLRP3, (c) caspase-1, and (d) ASC were normalized to *β*-actin. The results shown are the means ± SD of three independent experiments; ^#^*p* < 0.05 or ^##^*p* < 0.01 compared with the Con group; ^*∗*^*p* < 0.05 or ^*∗∗*^*p* < 0.01 compared with the MSU group.

**Figure 5:**
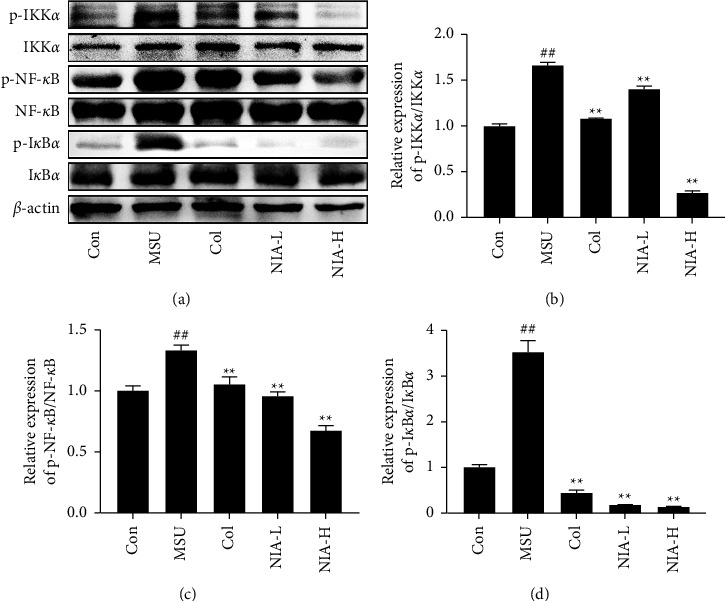
NIA inhibited the NF-*κ*B pathway in mouse joints. (a) Western blotting analysis of the NF-*κ*B pathway. The relative phosphorylation levels of (b) IKK*α*, (c) NF-*κ*B, and (d) I*κ*B*α* were normalized to the corresponding total protein. The results shown are the means ± SD of three independent experiments; ^#^*p* < 0.05 or ^##^*p* < 0.01 compared with the Con group; ^*∗*^*p* < 0.05 or ^*∗∗*^*p* < 0.01 compared with the MSU group.

To investigate the possible mechanism of NIA in treating AGA, changes in the NF-*κ*B/NLRP3 pathway were analyzed using western blotting. As demonstrated in Figure 4, MSU treatment significantly increased the expression of NLRP3, caspase-1, and ASC proteins, while these increases were reversed by NIA. As shown in Figure 5, MSU exhibited increased protein expression of phosphor-IKK*α*, phosphor-NF-*κ*B, and phospho-I*κ*B*α* without a change in total-IKK*α*, total-NF-*κ*B, and total-I*κ*B*α*. As expected, NIA plays a role in treating AGA by regulating the NF-*κ*B/NLRP3 pathway.

**Figure 6:**
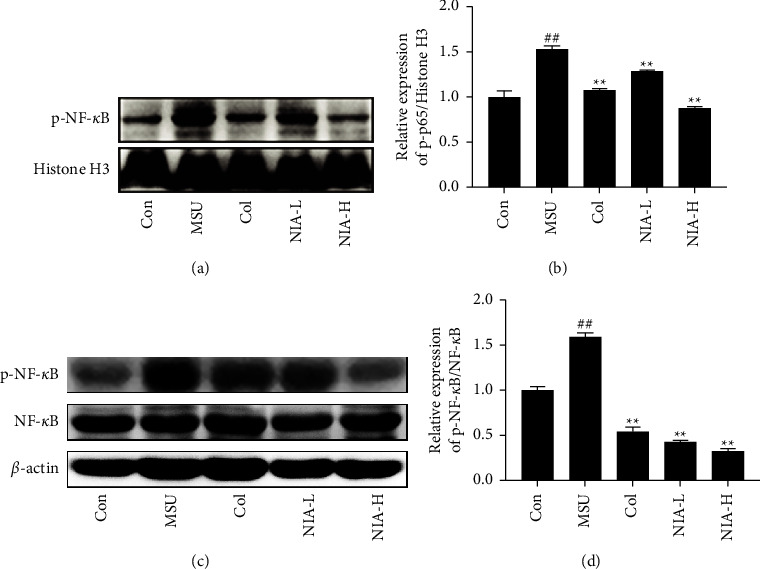
NIA reduced phospho-NF-*κ*B expression in the nucleus and cytoplasm. (a) Western blotting analysis of intranuclear phospho-NF-*κ*B. (b) The relative phosphorylation levels of phospho-NF-*κ*B were normalized to histone H3. (c) Western blotting analysis of intracytoplasmic phospho-NF-*κ*B. (d) The relative phosphorylation levels of phospho-NF-*κ*B were normalized to NF-*κ*B. The results shown are the means ± SD of three independent experiments; ^#^*p* < 0.05 or ^##^*p* < 0.01 compared with the Con group; ^*∗*^*p* < 0.05 or ^*∗∗*^*p* < 0.01 compared with the MSU group.

To further elucidate the roles of the NF-*κ*B/NLRP3 pathway in treating AGA, nuclear proteins and cytoplasmic proteins were isolated, and the expression of phospho-NF-*κ*B was detected. As illustrated in Figure 6, MSU significantly increased the expression of phospho-NF-*κ*B in the cytoplasm and nucleus, while Col and NIA both reverse the increased phospho-NF-*κ*B expression, with high doses of NIA being more effective than low doses of NIA. NIA inhibited NF-*κ*B from entering the nucleus to transcribe various inflammatory genes and reduced the synthesis of inflammatory cytokines. Together, these results suggested that NIA might inhibit AGA through the NF-*κ*B/NLRP3 pathway.

## 4. Discussion

AGA has been widely considered as one of the main types of gout that causes decreased quality of life, and its clinical manifestation is a self-limited attack of synovitis [20, 21]. With the upward trend in living standards, AGA has become a global health issue due to changes in dietary and lifestyle habits [22]. Currently, the drugs used to treat have severe side effects that make it possible to damage human health, necessitating to research agents that are efficacious and safe. Previous studies had demonstrated that astilbin ameliorates osteoarthritis by suppressing TLR4/MD-2 and NF-*κ*B signaling [23, 24]. Cai et al. [25] reported that astilbin also improved collagen-induced arthritis. Astilbin has the potential to improve AGA, and NIA is the stereoisomer of astilbin, so we speculate that NIA has a protective effect against AGA. After the MSU injection, the mice began to show decreased activity and joint swelling. The lesion severity of NIA-treated mice began to significantly decrease 2 h after modeling, and this trend lasted until the end of the day-long measurement. Furthermore, NIA treatment resulted in a significant reduction in inflammatory cell infiltration. These results clearly suggested that NIA is a potential therapeutic candidate for the treatment of AGA.

The pathogenesis and mechanism of AGA are complicated. In addition to genetic and dietary factors, the increased production of inflammatory cytokines plays a crucial role in AGA pathogenesis [26, 27]. MSU crystals induce secretion of inflammatory cytokines, including TNF-*α*, IL-6, and IL-1*β* in the progressive phase of AGA [28]. TNF-*α*, IL-6, and IL-1*β* are involved in the activation and maintenance of inflammatory responses, which serve key roles in the development and sustenance of gouty arthritis [29–31]. The study showed that chaetocin inhibits IL-1*β* secretion, thereby providing gout relief [32]. Curcumin prevents acute gout attacks by inhibiting the secretion of MSU-induced inflammatory cytokines [33]. As in the abovementioned studies, our study indicated that NIA treatment decreased the expression of IL-6, TNF-*α,* and IL-1*β*. Therefore, we speculate that NIA reduced the severity of AGA by suppressing the increased inflammatory response.

The NF-*κ*B/NLRP3 pathway plays a central role in inflammation-related diseases, including gouty arthritis, and the blockade of the NF-*κ*B/NLRP3 pathway is now proven to be an effective strategy to prevent and ameliorate AGA [34, 35]. During AGA onset, the IKK complex is phosphorylated and then the NF-*κ*B inhibitory protein I*κ*B*α* is phosphorylated. It increases NF-*κ*B phosphorylation levels and stimulates NF-*κ*B translocation into the nucleus [36]. Ultimately, activation of NF-*κ*B induces gene expression of IL-1*β* and NLRP3, which would induce the production of inactive IL-1*β* precursors [37]. NLRP3 first binds ASC to form an inflammasome that subsequently cleaves procaspase-1 to form active caspase-1 and, following cleavage, caspase-1 cleaves pro-IL-1*β* to the active form of IL-1*β* [38]. In addition to IL-1*β*, the core of gouty inflammation, NF-*κ*B/NLRP3 pathway involves activation of cytokines from a variety of cell types, including TNF-*α* and IL-6 [39]. Our study agreed with the abovementioned studies that the expression of NF-*κ*B/NLRP3 pathway-related proteins was significantly increased by MSU crystals. NIA significantly inhibited the expressions of the above proteins, which suggested NIA ameliorated AGA via suppression of the NF-*κ*B/NLRP3 pathway.

Inhibition of the NF-*κ*B/NLRP3 pathway may reduce the release of inflammatory factors such as IL-1*β* and consequently improve AGA. Our findings are consistent with some but not all previous studies. Although both NIA and colchicine were found to decrease inflammatory factors and inhibited NF-*κ*B/NLRP3 pathway, NIA is slightly more potent than colchicine at inhibiting NF-*κ*B/NLRP3 pathway, and colchicine is more potent than NIA in inhibiting the release of inflammatory factors. Research has shown colchicine can inhibit AMPK pathways, the RhoA/Rho effector kinase (ROCK), and other inflammation-related pathways [10, 40]; NIA may be weaker in some inflammation-related pathways, which was lacking in this experiment. These results indicate that NIA could be an effective agent in AGA treatment, which might also be used as an adjuvant in combination with other traditional anti-AGA drugs.

## 5. Conclusion

The present study demonstrated in C57BL/6 mice that NIA could alleviate MSU-induced AGA. The specific regulatory mechanism may be through the inhibition of NF-*κ*B/NLRP3 pathway, which may reduce the levels of inflammatory factors (IL-1*β*, IL-6, and TNF-*α*) and the inflammatory response in AGA.

## Data Availability

The data used to support the findings of this study are available from the corresponding author upon request.
